# Maternal and Pediatric Health Outcomes in relation to Gestational Vitamin D Sufficiency

**DOI:** 10.1155/2015/501829

**Published:** 2015-12-06

**Authors:** Stephen J. Genuis

**Affiliations:** Faculty of Medicine, University of Calgary and University of Alberta, 2935-66 Street, Edmonton, AB, Canada T6K 4C1

## Abstract

Juxtaposed with monumental improvement in maternal-fetal outcomes over the last century, there has been the recent emergence of rising rates of gestational complications including preterm birth, operative delivery, and gestational diabetes. At the same time, there has been a burgeoning problem with widespread vitamin D deficiency among populations of many developed nations. This paper provides a brief review of potential health outcomes recently linked to gestational vitamin D deficiency, including preterm birth, cesarean delivery, and gestational diabetes. Although immediate costs for obstetric complications related to gestational vitamin D insufficiency may be modest, the short- and long-term costs for pediatric healthcare resulting from such gestational complications may be enormous and present an enduring burden on healthcare systems. With increasing evidence pointing to fetal origins of some later life disease, securing vitamin D sufficiency in pregnancy appears to be a simple, safe, and cost-effective measure that can be incorporated into routine preconception and prenatal care in the offices of primary care clinicians. Education on gestational nutritional requirements should be a fundamental part of medical education and residency training, instruction that has been sorely lacking to date.

## 1. Introduction and Background

In the early and mid-1800s, the maternal mortality rate in some European obstetrical clinics approached 1 in 5 women as a result of puerperal fever [1]. With the epic discovery of the origins of this “childbed fever” by Ignaz Philipp Semmelweis in the mid-19th century and the eventual knowledge translation of his simple hand washing technique into the clinical domain, rates of postpartum mortality eventually decreased [1]. Over the subsequent century, there continued to be monumental advances in many areas of Maternal-Fetal Medicine. Along with a profound decline in maternal mortality from 7.2 deaths per 1000 births in the early 1900s to 0.08 by the end of 2000 [2, 3], there was a concomitant decline in infant mortality from 96 to less than 7 deaths per 1000 live births [2, 3]. With ongoing research and study over the last decades, remarkable advances have continued to be made in the assessment and management of a variety of gestational and perinatal challenges. Despite much success, however, there are new and emerging concerns in the early 21st century within the field of Maternal-Fetal Medicine.

Along with an astonishing rise in the rate of cesarean delivery with attendant complications to the human microbiome [4, 5], we have witnessed a concerning escalation in preterm birth [6], a complication associated with higher rates of long-term physical and mental health problems in the offspring [6–8]. The Institute of Medicine (IOM) estimated the annual costs for the burden of morbidity, disability, and mortality associated with preterm birth in the United States to be at least $26.2 billion [9]. Furthermore, the costs associated with neonatal intensive care, healthcare now required by an increasing percentage of the newborn population [10], are staggering [11]. It is also evident that maternal complications do not necessarily stop with giving birth. Rates of serious obstetrical complications such as postpartum depression, for example, extract enormous personal cost and remain a serious and widespread problem [12].

In addition, there is increasing discussion in the literature about fetal origins of pediatric and adult disease [13, 14], resulting from potentially modifiable gestational determinants such as disordered maternal nutrition [15] and toxic exposures [16–18]. As this is a new area of study, however, the extent of sequelae associated with modifiable gestational determinants is yet unrecognized; it is thus not possible to assign precise costs associated with long-term outcomes. It is important, however, to explore and implement clinical approaches during the preconception and gestational period which address determinants of suboptimal outcomes in order to maximize the enduring health of mothers and their offspring.

## 2. Modifiable Gestational Determinants and Illness

With recent attention to epigenetic research, it is becoming apparent that virtually all disease, including affliction in the gestational period, is the result of the interaction between our genes and the environment [19]. In fact, rather than genetic predestination [20], recent evidence confirms that modifiable environmental factors appear to be responsible for 70–90 percent of clinical illness [21]. Yet within the environmental domain, there appear to be only two determinants which make up the environment sphere. (i) Are we getting what we need? (ii) Are we being exposed to things that are toxic? [19]. Accordingly, it appears that the bulk of human disease, including problems in pregnancy, is related to deficiency and toxicity [19].

Evidence in the obstetric literature appears to support this contention and provides opportunity to make advances with regard to maternal and fetal well-being. In fact, medical intervention and maternal education delivered prior to conception (preconception care) to secure nutritional adequacy and preclude toxic exposures are being extolled as the next frontier of maternal and child healthcare [31]. The March of Dimes, a nonprofit organization dedicated to the health of mothers and babies, suggests that “the [physician] must take advantage of every health encounter to provide preconception care and risk reduction before and between conceptions—the time when it really can make a difference” [32]. With the evident link between fetal determinants and later onset disease, measures to secure an optimal gestational environment can have a profound impact on maternal and pediatric health with enormous personal, social, and financial savings.

There is considerable attention in the literature to the direct link between assorted toxicants in pregnancy and adverse maternal and fetal outcomes [16]. Most recently, FIGO (The International Federation of Obstetrics and Gynecology) released a special communication highlighting the urgent need to address the issue of widespread toxicant exposure and bioaccumulation in reproductive aged women [18]. In addition, it is becoming increasingly apparent that various nutritional deficiencies are widespread and may have an enormous impact on subsequent maternal and fetal health. Increasing evidence appears to confirm that at no point throughout the life cycle is it more important to secure adequate nutrient intake than in pregnancy [26]. This fact, for example, accounts for the emphasis on folate sufficiency in early gestation [33] as well as the increased study into the outcomes related to gestational deficiency of required omega-3 fatty acids [34] and magnesium [35].

With the emerging evidence that vitamin D acts epigenetically in the regulation of over 2700 different genes [36], there has been much recent research exploring the widespread prevalence of vitamin D deficiency through the continuum of life, including the gestational and neonatal period. This paper is designed to review the literature findings about the enduring impact of gestational vitamin D sufficiency on maternal and pediatric health and well-being.

## 3. Methods

This brief review was prepared by assessing available medical and scientific literature from Medline as well as by reviewing several books, nutritional journals, conference proceedings, government publications, and nutrition related periodicals. Terms searched included gestational vitamin D, pregnancy and vitamin D, fetus and vitamin D, nutrition in pregnancy, as well as pediatric health and vitamin D. Relevant references found in these publications were also searched in order to glean pertinent information. A primary observation, however, was that limited scientific literature is available on the issue of gestational vitamin D insufficiency as it relates to long-term health outcomes.

The format of a traditional integrated narrative review was chosen as such reviews play a pivotal role in scientific research and professional practice in medical issues spanning different medical disciplines, in this case obstetrics, pediatrics, and general medicine. Furthermore, this type of publication approach seemed apposite when endeavoring to answer specific clinical questions in a field with limited primary study [37]. Finally, it was deemed that a traditional integrated review paper might be optimal when exploring a myriad range of health outcomes, both short and long term.

## 4. Clinical Relevance of Vitamin D Sufficiency in Reproductive Healthcare

The widespread clinical importance of determining the correlation between vitamin D levels and reproductive outcomes is evident. The medical literature has achieved general consensus that vitamin D levels throughout much of the globe, as reflected by population measurements of 25(OH)D_3_ levels, are generally inadequate [38]. About 2/3 of the population in northern climates are considered deficient with average 25(OH)D_3_ levels in one study of 67 nmol/L [39], well below the 120–150 nmol/L level that has recently been associated with preferred health [24] (Table 1). With such widespread deficiency, it is vital to determine whether or not low gestational levels of vitamin D are a significant determinant of reproductive and pediatric health outcomes.

The need for clarity on this issue has also been recognized because of disparity about recommended dosing among esteemed medical groups. While the Institute of Medicine (IOM) agrees that 4,000 IU of vitamin daily is allowable and nontoxic, their actual recommended daily intake has been limited to 600 IU daily in general and 400 IU/day during gestation [40, 41]. These IOM recommendations for required vitamin D intake have been put into serious question [42], however, as a significant statistical error has been identified in the way their recommendations were arrived at [43]. Accordingly, exploration of consensus findings on the clinical benefits of vitamin D supplementation is in order in all medical disciplines including reproductive healthcare.

## 5. Limitations of Vitamin D Research as Related to Gestational Outcomes

Although maternal-fetal outcomes in the presence of adequate gestational vitamin D are generally favorable as reported in the medical literature, some reports have been inconsistent and cast doubt on the link between gestational vitamin D sufficiency and health. Specifically, supplementation of vitamin D in pregnancy in some studies appears to suggest marked benefit while research in other publications does not appear to confer significant improvement in maternal-fetal outcomes [44]. For example, a systematic review and meta-analysis by Pérez-López et al. found that gestational vitamin D supplementation was associated with increased birth weight and birth length but, unlike some other research, was not associated with other beneficial maternal and neonatal outcomes such as reductions in preeclampsia, gestational diabetes, small for gestational age infants, preterm birth, or rates of cesarean delivery [44]. The apparent disparity between findings in various studies has caused some to reflexively conclude that vitamin D status in pregnancy is irrelevant to maternal-fetal outcomes.

Studies on reproductive outcomes related to vitamin D supplementation, however, are inherently plagued by a number of common confounders which cloud the picture. It is important to realize that vitamin D status is very different from whether or not someone is consuming vitamin D supplementation. Many factors may affect the resultant status of vitamin D in the body (as reflected by measurement of 25(OH)D levels) after ingested supplementation. Dosing of supplements, body weight, levels of various toxicants, and individual metabolism can all be factors in consequent vitamin D indices after supplementation. Many of the recent publications challenging the efficacy of gestational vitamin D sufficiency have been meta-analyses which attempt to synthesize diverse data from numerous observational and supplementation studies which do not necessarily incorporate individual differences in these central determinants.

Specific concerns about several vitamin D meta-analyses can account for the varying outcomes reported from this type of research. (i) There is wide heterogeneity of studied populations with variations in vitamin D supplement dosing, geophysical location, social and dietary conditions, and other factors in studied groups [45]. Supplementation at varying doses (e.g., 400 IU/day versus 4000 IU/day), for example, may achieve remarkably different levels of serum 25(OH)D and thus different outcomes. (ii) Commencement of supplementation at differing times during the gestation may miss critical periods when vitamin D may play a pivotal role. (iii) Different types of vitamin D (vitamin D_2_ versus vitamin D_3_) have different physiological impact. And (iv) various methodological concerns are evident [46], such as the lack of standardized assays.

In addition, it is well recognized in healthcare that regardless of how compelling the evidence on a specific scientific or medical issue, introduction of doubt can be a potent impediment to the implementation of effective public health and clinical measures [47, 48]. Accordingly, a critical appraisal of such meta-analyses is in order to achieve an accurate perspective on the efficacy of gestational vitamin D supplementation.

## 6. Gestational Vitamin D Status and Obstetrical Outcomes

The list of adverse gestational outcomes in pregnancy associated with vitamin D insufficiency continues to mount. Early in pregnancy, for example, an increased risk of first trimester miscarriage has been linked to inadequate maternal vitamin D levels [49]. Interestingly, one study demonstrated that nearly half the women assessed with habitual miscarriage were found to have 25(OH)D levels below 75 nmol/L [50]. This research found that lower vitamin D levels were associated with immune dysregulation in a number of ways, including differences in indices involving natural killer cells, various cytokines, and certain regulatory proteins, when compared to those with sufficient vitamin D levels [50]. The authors of this study also noted that women with lower vitamin D levels had higher degrees of various autoantibodies including antiphospholipid antibody [50], a clinical state that has been associated with fetal death, recurrent early miscarriage, preeclampsia, and placental insufficiency [51].

A number of papers have confirmed an increased risk of developing gestational diabetes in those with inadequate vitamin D levels [52, 53]. Vitamin D sufficiency in pregnancy appears to be related to improved insulin levels, as well as better glucose regulation as reflected by HbA1c levels [54]. As pregnancies complicated by gestational diabetes present risks for assorted adverse sequelae, efforts to avoid dysregulated sugar control in pregnancy are worthwhile. Obesity presents a confounding influence in this discussion, however, as a greater BMI is associated with lower vitamin D levels as well as greater insulin resistance and risk for gestational diabetes. Numerous studies have also correlated low levels of vitamin D with the development of preeclampsia [52, 53, 55, 56], perhaps through immune mechanisms involving antiphospholipid antibody [51], and/or inflammatory mechanisms involving cytokines [56].

Of particular significance is the reality that preterm birth before 37 weeks of gestation remains the leading cause of neonatal morbidity and mortality [57]. Escalating evidence in the literature confirms a protective association between maternal vitamin D sufficiency and the incidence of preterm birth [58–60]. A recent study found that the rate of occurrence of preterm birth appeared to be inversely parallel to the maternal serum 25-hydroxyvitamin D levels [58]. The authors report that the incidence of preterm birth at less than 37 weeks of gestation was (i) 11.3%, (ii) 8.6%, and (iii) 7.3% among mothers with respective serum 25-hydroxyvitamin D levels of (i) less than 50, (ii) between 50 and 74.9, and (iii) 75 nmol/L or greater. Another study found that infants born before 32 weeks of gestation were 2.4 times more likely to have vitamin D levels below 50 nmol/L when compared with those born after 32 weeks of gestation [61].

Other gestational issues also appear to be influenced by maternal vitamin D levels. Vitamin D insufficiency, for example, has also been correlated with maternal periodontal disease [62], a higher likelihood of small for gestational age infants [63, 64], and an increased risk of bacterial vaginosis [65].

As well as individual studies, systematic reviews exploring association between vitamin D sufficiency and health outcomes have also been illuminating. A systematic review and meta-analysis of 24 studies suggested that low maternal vitamin D levels in pregnancy may be associated with an increased risk of small for gestational age infants, as well as being linked to preeclampsia, gestational diabetes, and preterm birth [66]. Another systematic review and meta-analysis published in the* British Medical Journal* also linked vitamin D insufficiency with an increased risk of gestational diabetes, preeclampsia, and small for gestational age infants [65].

Vitamin D status also appears to influence modes of delivery. Vitamin D deficiency has been linked to increased odds of primary cesarean delivery [67] as well as a higher likelihood of emergency cesarean section [68]. In one study, women with vitamin D levels below 37.5 nmol/L were almost four times as likely to require a primary cesarean delivery as women with higher levels [67]. Through the evolving work on the Human Microbiome Project, it has recently been found that the infant's journey through the birth canal is instrumental in shaping a healthy microbiome, a feature which appears to be a determinant of subsequent health and which may be compromised by cesarean delivery [69]. Accordingly, efforts to diminish the high rates of Cesarean delivery are warranted.

Gestational vitamin D status also appears to influence outcomes beyond the pregnancy and delivery. A very challenging problem for many new mothers is postpartum depression. There is escalating evidence in general that low vitamin D levels are correlated with higher risk for a variety of mental health problems including depressive illness [70], as vitamin D is known to play an important role in activating genes that release neurotransmitters such as dopamine and serotonin [70, 71]. Intervention to normalize levels of vitamin D appears to be successful in restoring mental health [72]. In particular, in relation to maternal health, a recent study published in the* British Journal of Obstetrics and Gynecology* reported that serum 25[OH]D levels in women with no postpartum depression were significantly higher than those in women suffering with postpartum depression [73].

## 7. Gestational Vitamin D Status and Later Life Outcomes

Although research into fetal origins of disease in later life remains in its infancy, there is increasing suspicion that gestational nutritional sufficiency may be a determinant of health in later life. For example, preliminary evidence suggests that insufficient levels of prenatal vitamin D may be a factor in the development of autism [74] and lower respiratory infections [75]. A Spanish study to this end found that mothers who had gestational vitamin D levels above 75 nmol/L had offspring with a one-third lower rate of acute respiratory tract infections during the first year of life [76].

A recent body of work has begun to suggest that lower gestational vitamin D levels may also be associated with higher rates of pediatric atopic disease [77], food sensitivities [78], atopic dermatitis [79], eczema [80], asthma [81], impaired lung function [82], allergic disease [83], and other conditions frequently characterized by a hypersensitive immune state. It appears that fetal vitamin D levels may play a modulating role in immune functions involved in atopic disorders. As hypersensitivity outcomes may also be seen in those children born to mothers contaminated with assorted xenobiotics in pregnancy [84–89], however, it is not known whether the immune dysregulation and hypersensitivity may be the consequence of a primary gestational insufficiency of vitamin D, or whether various chemical toxicants might play a role by impairing vitamin D uptake, renal synthesis, and assimilation [25, 90] while at the same time inducing immune compromise and hypersensitivity through other mechanisms [91].

Maternal vitamin D status during gestation also appears to have influence on many other health indices in the future of the offspring. For example, gestational vitamin D status directly correlates with subsequent whole-body and lumbar spine bone mineral content in progeny at 9 years of age [92]. Furthermore, an interesting cohort study correlating maternal vitamin D deficiency at 18 weeks of pregnancy and health outcomes of progeny found that gestational vitamin D deficiency was associated with impaired lung development in 6-year-old offspring, neurocognitive difficulties at age 10, increased risk of eating disorders in adolescence, and lower peak bone mass at 20 years [93]. The authors state that “vitamin D may have an important, multifaceted role in the development of fetal lungs, brain, and bone” [93]. Finally, gestational vitamin D levels may impact adult health as there is early evidence that vitamin D sufficiency in pregnancy may have a protective role in the development of adult onset multiple sclerosis [94].

## 8. Economic Burden of Gestational Vitamin D Deficiency

The economic impact of vitamin D deficiency as it relates to maternal-fetal health is difficult to objectively quantify as insufficient evidence has accumulated thus far on the totality of short- and long-term sequelae of vitamin D insufficiency. Furthermore, current appraisals tend to underestimate the extent of the required resource utilization for specific conditions associated with vitamin D insufficiency. For example, cost estimates for the immediate care involved with the increase in cesarean delivery rates can be calculated, but these do not at all take into account unexpected surgical complications [95] that may arise in the future or the enormous potential cost impact from enduring microbiome changes resulting from operative delivery [69, 96]. The reality is that adverse gestational complications do not end with the pregnancy and can result in morbidity and resource utilization extending throughout the life of the offspring.

This is also demonstrated by the challenge of preterm birth, as premature birth results in significant morbidity, mortality, healthcare utilization, and associated costs starting in infancy and extending for years to come [97]. This can be quite a burden on national healthcare systems. In Canada, for example, the estimated additional 10-year cost to care for the children born prematurely each year is hundreds of millions of dollars [98]. Furthermore, many health problems sustained by children born prematurely continue far beyond their tenth birthday with a consequent and sometimes ongoing economic burden placed on health, education, and social service resources. With regard to economic challenges associated with vitamin D insufficiency, suffice it to say that there are considerable costs potentially associated with gestational vitamin D insufficiency [99] as well as corresponding benefits and cost savings resulting from inexpensive supplementation with this essential nutrient [99].

## 9. Conclusion

There is escalating attention in the scientific literature to the association between myriad nutrients and health outcomes [100, 101]. Training in clinical nutritional biochemistry, nonetheless, still remains woefully inadequate or nonexistent in most medical education programs [102, 103]. As a result, there are ongoing calls of late for curriculum revision to incorporate practical training in clinical nutrition [103, 104]. It is apparent that training is required to establish clinical competency in (i) understanding of the role of various nutrients in human health, (ii) how to assess nutritional biochemistry in patients, and (iii) and how to intervene to secure nutrient sufficiency for individuals and population groups

With the mounting evidence of several health sequelae associated with gestational vitamin D deficiency, the value of preconception education and care by health providers and public health bodies to secure vitamin D sufficiency throughout gestation is evident. As pregnant women [105], particularly those with dark skin [106], are at considerable risk for experiencing vitamin D insufficiency [39], it is important to have a high index of suspicion and to effectively preclude, assess for, and manage vitamin D inadequacy, as would be done with other biochemical irregularities.

Although (to the author's knowledge) there are no specific target levels for 25(OH)D during the various stages of pregnancy that correlate with optimal results in relation to maternal and pediatric health outcomes, some authors have made recommendations for supplemental levels that appear to be safe and effective. These recommendations range from 600 IU/day from the Institute of Medicine [41] to 2000 IU/day from the Canadian Pediatric Society [107], to 4000 IU/daily from various researchers who have concluded that the latter dose is both efficacious and safe [26–30]. One researcher has suggested that the dietary requirement during pregnancy and lactation may actually be as high as 6000 IU/day [108], but most researchers have concluded, with our current knowledge, that a supplemental vitamin D intake of 4000 IU/day is optimal [30]. As discussed, individual vitamin D indices can be influenced by various determinants despite specific levels of supplementation; it is thus the author's recommendation that a personalized medical approach be taken via individual screening for 25(OH)D as a routine part of preconception and prenatal care.

With evidence that a major proportion of the adult population [38], particularly in northern climates [39], is potentially deficient in vitamin D, it appears that, at minimum, one out of every few expectant mothers will have inadequate levels of this essential nutrient. With the recognition that vitamin D plays an essential role in myriad genes that encode for health and well-being in the offspring, it behooves the medical and public health community to endeavor to secure vitamin D adequacy in the gestational period. The ongoing personal health burden associated with gestational vitamin D insufficiency throughout many parts of the world has the potential to be ameliorated considerably by straightforward educational and healthcare measures in the preconception and prenatal period to secure vitamin D sufficiency throughout pregnancy. It is time for maternity health providers to be apprised of the potential for improved and enduring health and well-being associated with inexpensive measures to secure vitamin D nutritional adequacy during gestation, the most vulnerable time in the life cycle of the developing child.

## Figures and Tables

**Table 1:** Optimal adult levels of vitamin D (as reflected by 25(OH)D levels) from different sources.

Source	Vitamin D level (nmol/L)	Vitamin D level (ng/mL)
Holick (2010) [22]	100–150	40–60
Endocrine Society (2011) [23]	At least >75, aim for 100	At least >30, aim for 40
Amrein et al. (2014) [24]	120–150	48–60
Schwalfenberg and Genuis (2015) [25]	120–150	48–60

No consensus on a specific optimal 25(OH)D level in pregnancy has been achieved.

Emerging agreement that supplemental vitamin D_3_ at a dosage of 4000 IU/day throughout pregnancy may be safe and effective [26–30].
